# Association between passive smoking and mental distress in adult never-smokers: a cross-sectional study

**DOI:** 10.1136/bmjopen-2016-011671

**Published:** 2016-07-29

**Authors:** Rui Wang, Peng Zhang, Xin Lv, Chunshi Gao, Yuanyuan Song, Zhijun Li, Yaqin Yu, Bo Li

**Affiliations:** Department of Epidemiology and Biostatistics, Jilin University School of Public Health, Jilin, China

**Keywords:** Chinese never-smokers, mental stress, passive smoking, GHQ-12

## Abstract

**Objective:**

Many studies have suggested exposure to secondhand smoke (SHS) is a risk factor for various somatic diseases, but only few studies based on small sample size or specific groups have explored the association between passive smoking and mental distress. We performed this study to examine the relationship between passive smoking and mental distress in adult never-smokers of north-east China.

**Methods:**

Multistage, stratified random cluster sampling design was used in this cross-sectional study in 2012. A total of 12 978 never-smokers from Jilin, north-east China, were included. Data on passive smoking and baseline characteristics were collected by face-to-face interviews. The 12-item General Health Questionnaire (GHQ-12) was used to measure mental health status. Rao-Scott χ^2^ tests were used to compare the prevalence between different groups; multivariable logistic regression was used to assess the association between passive smoking and mental distress, and Spearman rank analysis was employed to assess the correlation between passive smoking and GHQ-12 scores.

**Results:**

The estimated prevalence of mental distress among never-smokers in Jilin province is 24.5%, and the estimated prevalence of passive smoking among the mental distressing group is 65.0%. After adjusting for gender, age, region, body mass index (BMI), occupation, marriage, education, drinking status and family monthly income per capita, passive smoking conferred a risk for mental distress (adjusted OR=1.26, 95% CI 1.13 to 1.40). A high proportion of adults, especially women, were passive smokers at home, but for men, passive smoking was more common at workplace. The more frequently participants exposed to SHS, the higher GHQ-12 scores they got.

**Conclusions:**

Passive smoking is an important risk factor for mental distress in never-smokers of Jilin province, which reminds Chinese government of increasing the awareness of public health and take measure to prevent SHS, especially with regard to SHS exposure at home and workplace.

**Strengths and limitations of this study**

- To the best of our knowledge, this study is the first face-to-face community-based investigation that examined the relationship between passive smoking and mental distress among adult never-smokers in north-east China, which contains the largest sample size.
- Multistage, stratified random cluster sampling design was used in this study to make the data representative.
- The main limitation of this study was the use of self-reported data and the nature of cross-sectional data, which may lead to recall and reporting biases.

## Introduction

Tobacco smoking is a major global public health issue, which proved to be an important risk factor for several systemic inflammation diseases.^1–3^ At present, there are more than 1 billion smokers all over the world. The WHO predicts that smoking will contribute to more than 8 million deaths around the world in 2030.^4^ In China, there are ∼300 million smokers nationwide, which occupies about one-third of all smokers worldwide. The status of passive smoking in China is also serious: about 51.9% of non-smokers were frequently exposed to secondhand smoke (SHS) in 2002 and 72.4% in 2010.^5^ Presently, there are still 740 million Chinese people exposed to SHS, including 180 million children under the age of 15.^6^ Jilin is the second-largest province in north-east China; the overall prevalence of passive smoking among non-smoking women in Jilin province was 60.6% in 2012.^7^ The high prevalence of passive smoking has been a major focus in China.

SHS is an artificial environmental risk factor, associated with various adverse health outcomes,^8–10^ which may confer significant economic and medical burdens to China. Mental health in China is also a great concern given the large number of patients and huge social and economic costs. The estimated prevalence of adult mental disorder in China is about 17.5%, and there are over 170 million adults having one or more types of mental disorder.^11^ Various individual and environmental factors influence mental distress. It is well established that active smoking is associated with mental illness,^12^ but the relationship between passive smoking and mental distress remains in dispute.^13–16^ Furthermore, in developing countries, information on the association between passive smoking and mental distress is scarce. Although several studies have suggested a significant linkage between mental distress and passive smoking, research based on Chinese population was so limited that only one study was found whose finding supported the health hazards of SHS exposure.

Examining the relationship between passive smoking and mental distress may contribute to ameliorating the status of public psychology. Therefore, a robust result based on large sample size including both genders and all age groups is needed. To evaluate the relationship between passive smoking and mental distress, we investigated mental distress prevalence among adult never-smokers in Jilin province. We collected data on baseline and passive smoking by face-to-face survey, and mental distress was measured by the12-item General Health Questionnaire (GHQ-12).

## Methods

### Study design and population

Data of this study were acquired from the Project on Present Situation and Change Forecast of Disease Spectrum in Jilin province of China. This survey was a face-to-face, cross-sectional interview among individuals aged 18–79 years, which was conducted in 2012. We used multistage, stratified cluster sampling method to select all the participants. People from 9 regions (Changchun, Jilin, Siping, Liaoyuan, Tonghua, Baishan, Songyuan, Baicheng and Yanbian), 32 districts or counties, 95 towns or communities and 45 units in Jilin province were selected. The detailed stratifying process has been published previously.^17^ We recruited 23 050 adult participants in total, and 21 435 participants completed the survey (response rate: 84.9%). A total of 12 978 (60.5%) adult never-smokers were included in our study. This study was approved by the Ethics Committee of Jilin University School of Public Health, and written informed consents were obtained from all the participants in the survey.

### Data collection and definition of major variables

A total of 116 trained investigators conducted the survey using a structured questionnaire. The questionnaire included the information on participants' sociodemographic characteristics, passive smoking status and other related information on health. Physical examination was conducted for all the participants, including height, weight, fasting blood glucose, blood pressure and blood lipid, by standard instruments. Smoking status was classified into three groups: current, former and never. Current smokers were defined as those who consumed any kind of tobacco products at the time of the interview, former smokers were those who smoked in the past but had hitherto given up smoking for over 3 months and participants who reported never having smoked 100 cigarettes were defined as never-smokers.^18^ Only never-smokers were included in this study. Participants who answered ‘yes’ to the following questions were defined as passive smokers: ‘During the past week, were you exposed to SHS for more than 30 minutes once?’ or ‘During the past week, did anyone smoke around you for more than 30 minutes once?’ Then the following questions were asked to collect exposure frequency and places: ‘How often were you exposed to SHS?’ and ‘Where were you exposed to SHS regularly? Home, workplace, restaurant, entertainment place or other places?’ For students, ‘workplaces’ included classrooms and libraries.^19^

The dependent variable in this study is mental distress, which was measured using the GHQ-12. The GHQ-12 is a standard measure of mental distress which has been used to detect the presence of anxiety and depression in various populations.^20^ ^21^ The external validity and internal sensitivity of the instrument are generally adequate in the Chinese population.^22^ ^23^ This survey has a four-point response scale (0–3) transformed into a score (0–0–1–1);^24^ the total score of 12 items is between 0 and 12. A higher score indicates more psychological problems. According to the total score of the GHQ-12, the cut-off score for mental distress has been established at ≥3.^24^ In order to ensure the privacy of participants, the interviews were conducted privately in participants' homes or at designated quiet places such as consultation room of health centre. Each interview took ∼30 min. Interviewers would explain to participants if they were confused about any items on the questionnaire.

### Statistical analyses

Data were analysed using the SPSS (V.22.0; IBM Corp, Armonk, New York, USA) based on the complex sampling design. Poststratification adjustment was carried out to make the sample representative of the population in Jilin province.^17^ We also compared region, age, body mass index (BMI), marriage, occupation, education, drinking status and family monthly income per capita between distressing and non-distressing groups. We used Rao-Scott χ^2^ tests to compare the prevalence of mental distress in both groups. Logistic regression model was employed to calculate ORs and 95% CIs. To adjust for potential confounding effects, multivariable logistic regression analyses were conducted after adjusting for sociodemographic characteristics. Spearman rank analysis was also employed to assess the correlation between status of passive smoking and GHQ-12 scores. A p<0.05 was considered to be statistically significant.

#### Result

In this cross-sectional study, we recruited 23 050 participants in total; 21 435 participants completed the interview (response rate: 84.9%). After excluding current smokers (n=6822), ever-smokers (n=1557) and participants for whom data of smoking status were missing (n=78), 12 978 participants were included in our study. Of these participants, 7943 (estimated rate: 61.2%) participants reported exposure to SHS, and 3180 (estimated rate: 24.5%) participants had mental distress. As table 1 lists the baseline characteristics of distressing and non-distressing groups, sociodemographic characteristics differed almost in all aspects except for age groups.

**Table 1:** Baseline characteristics of the distressing and non-distressing groups

	Distressing group (n=3180)		Non-distressing group (n=9798)		
Characteristics	n	Per cent	n	Per cent	p Value
Region					p<0.001
Rural	1575	49.5	4079	41.6	
Urban	1605	50.5	5719	58.4	
Gender					p<0.001
Male	723	22.7	3154	32.2	
Female	2457	77.3	6644	67.8	
Age (years, mean (SD))	46.7±13.7		46±13.3		0.941
BMI					p<0.001
Normal	1680	52.9	4737	48.3	
Underweight	201	6.3	562	5.7	
Overweight	920	28.9	3081	31.4	
Obesity	379	11.9	1417	14.5	
Education					p<0.001
Primary school and below	908	28.5	1983	20.2	
Junior middle school	941	29.6	2836	28.9	
Senior middle school	761	23.9	2614	26.7	
Undergraduate and above	570	17.9	2364	24.1	
Occupation					p<0.05
Intelligence	739	23.2	2794	28.5	
Manual	1684	53.0	4684	47.8	
Retired	247	7.8	1088	11.1	
Others	509	16.0	1232	12.6	
Marriage status					p<0.001
Married	2470	77.7	7923	80.9	
Single	497	15.6	1411	14.4	
Divorced/separated	49	1.5	128	1.3	
Widowed	164	5.1	336	3.4	
Drink					p<0.05
Yes	600	18.9	2021	20.6	
No	2580	81.1	7778	79.4	
Family monthly income per capita (RMB)					p<0.001
<500	663	20.8	1312	13.4	
500–999	572	18.0	1638	16.7	
1000–1999	1083	34.1	3523	36.0	
2000–2999	573	18.0	2165	22.1	
>3000	289	9.1	1160	11.8	

Values for certain characteristics may not be equal to the total number of participants in the distressing and non-distressing groups because of missing data and complex weighted computation.

BMI, body mass index.

Table 2 presents the distribution of passive smoking among never-smokers in Jilin province (2012). Several prior studies have suggested there was a difference in exposure to SHS between both sexes; we also presented analyses separately for men and women. Overall, 5524 (60.7%) never-smoking women and 2419 (62.4%) never-smoking men described themselves as passive smokers. The frequency distributions of passive smoking in women were higher than that in men. A week before this investigation there were 1030 (26.6%) never-smoking men reported 1–3 days per week passive smoking exposure, and 2389 (26.2%) never-smoking women reported daily passive smoking exposure. Of the places at which participants were exposed to SHS, ∼43.2% female never-smokers reported their exposure at home, and 35.2% male never-smokers were exposed to passive smoking at workplace.

**Table 2:** Percent distribution of passive smoking status among adult never-smokers in both genders

	Male (n=3877)*		Female (n=9101)*	
	n	Per cent	n	Per cent
No passive smoking†	1458	37.6	3577	39.3
Passive smoking (times/week)	2419	62.4	5524	60.7
1–3	1030	26.6	1870	20.5
4–6	703	18.1	1274	14.0
>6	686	17.7	2380	26.2
Source of passive smoking				
Home	642	16.6	3932	43.2
Workplace	1366	35.2	1412	15.5
Restaurant	350	9.0	469	5.1
Entertainment place	569	14.7	781	8.6
Others	140	3.6	192	2.1

*Complex weighted computation was used in the statistical analysis.

†Exposure to secondhand smoke at least once per week for ≥30 min per occasion at home or public places.

Table 3 shows the association between passive smoking and mental distress. The proportion of passive-smoking participants (65.0%) in the distressing group was significantly higher than that in the non-distressing group (60.0%, p<0.05). The crude OR of distressing group was 1.24 (95% CI 1.12 to 1.37). After adjusting for sociodemographic characteristics—gender, age, region, BMI, occupation, marriage, education, drinking status and family monthly income per capita—the OR changed to 1.26 (95% CI 1.13 to 1.40). We also observed a positive dose–response association between exposure frequency and risk of mental distress in crude OR (1–3 times per week: OR=1.06; 4–6 times per week: OR=1.31; over 6 times per week: OR=1.38) compared with the reference group (no passive smoking). However, after we adjusted for the sociodemographic characteristics mentioned above, this trend was weakened.

**Table 3:** Prevalence of passive smoking distress risk

	Distressing group (n=3180) n (%)	Non-distressing group (n=9798) n (%)	Crude OR (95% CI)	Adjusted* OR (95% CI)
No passive smoking	1113 (35.0)	3922 (40.0)	1	1
Passive smoking (times/week)	2067 (65.0)	5876 (60.0)	1.24 (1.12 to 1.37)	1.26 (1.13 to 1.40)
1–3	668 (21.0)	2231 (22.8)	1.06 (0.91 to 1.22)	1.14 (0.98 to 1.32)
4–6	537 (16.9)	1440 (14.7)	1.31 (1.12 to 1.53)	1.37 (1.12 to 1.60)
>6	862 (27.1)	2205 (22.5)	1.38 (1.21 to 1.57)	1.30 (1.14 to 1.49)

*Adjusted for gender, age, region, BMI, occupation, marriage, education, drinking and family monthly income per capita.

BMI, body mass index.

**Figure 1:**
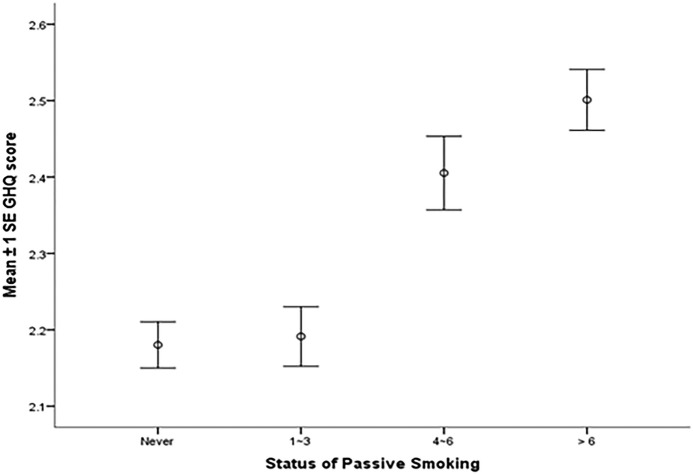
The GHQ-12 scores of four passive smoking groups. GHQ-12, 12-item General Health Questionnaire.

Figure 1 indicates different passive smoking statuses correlate with the GHQ-12 scores (r=0.074, p<0.001; the result of collinearity diagnostics is presented in the online supplementary file). The more frequently participants exposed to SHS, the higher GHQ-12 scores they got (never: 2.18±2.20; 1–3 times/week: 2.19±2.02; 4–6 times/week: 2.41±2.09; >6 times/week: 2.50±2.20).

10.1136/gutjnl-2015-311146.supp1 Supplementary table

## Discussion

Our study explored the association between passive smoking and mental distress incidence among adult never-smokers in Jilin, north-east China. We found SHS intensity represented a noticeable environmental factor contributing to adult mental distress, and as the frequency of exposure to SHS increased, the risk of mental distress increased. There were several possible explanations about the relationship between exposure to passive smoke and mental distress among adult never-smokers in north-east China. Some research studies have reported that individuals who are in stressful state for a long time may have a variety of psychological problems,^25^ ^26^ and a positive lifestyle (incorporating physical exercise and healthy eating habits) was proved to be linked with a low risk of mental distress.^27^ ^28^ A previous study also demonstrated that Chinese traditional Confucianism may affect people not to express their feelings directly or take measures to change the unpleasant environment.^29^ The traditional thought of Confucianism advocates the doctrine of the mean, the harmonious way of thinking. Remaining neutral in unpleasant environment has always been considered as a kind of traditional virtue. When people are faced with unpleasant surrounding environment such as exposure to SHS, most of them select patience rather than prevention. Moreover, there were some researchers who illustrated the association between passive smoking and mental distress from the aspect of hormone. They found that exposure to SHS can increase levels of corticotrophin-releasing hormone (CRH) and adrenocorticotropic hormone (ACTH),^30^ ^31^ which can affect people's mood, cognition and behaviour gradually.^32^

Our results suggest exposure to SHS may exacerbate mental distress, which are consistent with conclusions from previous research.^24^ ^33^ In other surveys, especially those focused on never-smokers, Kim NH *et al*^34^ found exposure to SHS may be associated with mental health problems such as depression and stress in Korean adults, and Asbridge *et al* found people who were exposed to SHS were more likely to report high mental stress in Canadian adults. Furthermore, two European researches have examined the association between salivary cotinine level and risk of psychiatric hospital admissions; they found a positive cotinine level–psychological distress relationship was apparent.^13^ ^33^ Moreover, a Chinese study also supported the mental health hazards of SHS exposure, which suggested SHS exposure prevalence at workplaces and public places was at a high level.^6^ However, a UK-wide, general population-based study showed discrepancies with our study that has shown no associations between SHS exposure and mental distress.^13^ Differences in study participants, mental health assessment and analysis methods may account for the conflicting evidence, although they did find a clear relationship between direct SHS exposure and mental health. Thus, there is a need for further research in this growing field of study.

Our study also confirmed exposure to SHS in Jilin province was very common. Over 60% never-smokers in this study reported exposures to passive smoke. A high proportion of Chinese, especially women, were passive smokers at home, but for men, passive smoking was more common at the workplace. Although China has taken measures to ban smoking at public areas such as railway stations, hospitals and schools, but there are still a relatively large number of people including children that are exposed to SHS at home.^35^ Two prior studies also reported similar results.^36^ ^37^ There is no doubt that exposure to SHS pollution has been a severe public health problem in China.

SHS exposure and mental distress are major public health problems. Our study provided new evidence on the association between SHS exposure and mental distress among this representative sample of Chinese adults. It is well known that smoke-free policies are the most effective way to reduce exposure to tobacco smoke in public areas; therefore, government and health departments should focus on banning smoking in public areas and also at home. Further, smoke-free policies will have a positive influence on smoking in households. Moreover, public education campaigns also need to improve awareness of the health damage associated with SHS.

The main limitation of our study is using the self-reported data of passive smoking and GHQ-12 questionnaire. Self-reported data and the nature of cross-sectional data may lead to recall and reporting biases, which may have affected the accuracy of the results. A lack of biological measures and longitudinal study may also degrade the precision of our result. In addition, in an interview setting, persons may give more socially desirable answers compared to a questionnaire. To control the false negatives, some previous research eliminated participants scoring below 3 on the GHQ-12 from their study.^38^ However, as a result of the low educational background (rate of primary school and below: 22%), self-administered questionnaire was not feasible in this survey. The result may be influenced by the false negatives, although we have selected private places and provided enough time for the interview. Nevertheless, our study also had several strengths. First, to the best of our knowledge, this is the largest face-to-face investigation in north-east China; 12 798 never-smokers were included in our study. Second, the use of complex sampling design and complex weighted computation improved the representation of the sample. Finally, including never-smokers only allowed us to distinguish the effects of passive smoking compared with active smoking.

## Conclusion

According to the results of our study, we demonstrated a significant association between mental distress and passive smoking in an adult never-smoking sample, which indicates that passive smoking is an important risk factor for mental distress development. We suggest that passive smoking in Jilin province represents a serious public health issue, especially with regard to SHS exposure at home and workplace. Consequently, the Chinese government should pay more attention to popularise the health damage of smoking and actively pursue measures to increase awareness of the health hazards associated with passive smoking.

## References

[1] Uruska A, Araszkiewicz A, Uruski P Higher risk of microvascular complications in smokers with type 1 diabetes despite intensive insulin therapy. Microvasc Res 2014;92:79–84. 10.1016/j.mvr.2014.01.002 24423616

[2] Feng D, Liu T, Su DF The association between smoking quantity and hypertension mediated by inflammation in Chinese current smokers. J Hypertens 2013;31:1798–805. 10.1097/HJH.0b013e328362c21a 24036901

[3] Zhang P, Wang R, Li Z The risk of smoking on multiple sclerosis: a meta-analysis based on 20,626 cases from case-control and cohort studies. PeerJ 2016;4:e1797 10.7717/peerj.1797 27014514 PMC4806598

[4] Li Z, Yao Y, Han W Smoking prevalence and associated factors as well as attitudes and perceptions towards Tobacco Control in Northeast China. Int J Environ Res Public Health 2015;12:8606–18. 10.3390/ijerph120708606 26206569 PMC4515736

[5] Yang GH, Ma JM, Liu N [Smoking and passive smoking in Chinese, 2002]. Zhonghua Liu Xing Bing Xue Za Zhi 2005;26:77–83. 15921604

[6] Yang T, Cao C, Cottrell RR Second hand smoke exposure in public venues and mental disorder: a representative nationwide study of China. Tob Induc Dis 2015;13:18 10.1186/s12971-015-0046-7 26185493 PMC4504412

[7] Li Z, Yao Y, Yu Y Prevalence and associated factors of passive smoking among Women in Jilin Province, China: a cross-sectional study. Int J Environ Res Public Health 2015;12:13970–80. 10.3390/ijerph121113970 26529002 PMC4661627

[8] Li N, Li Z, Chen S Effects of passive smoking on hypertension in rural Chinese nonsmoking women. J Hypertens 2015;33:2210–14. 10.1097/HJH.0000000000000694 26259123

[9] Eze IC, Schaffner E, Zemp E Environmental tobacco smoke exposure and diabetes in adult never-smokers. Environ Health 2014;13:74 10.1186/1476-069X-13-74 25253088 PMC4192739

[10] Okoli CT, Kelly T, Hahn EJ Secondhand smoke and nicotine exposure: a brief review. Addict Behav 2007;32:1977–88. 10.1016/j.addbeh.2006.12.024 17270359

[11] Qian J Mental health care in China: providing services for under-treated patients. J Ment Health Policy Econ 2012;15:179–86. 23525836

[12] Taylor G, McNeill A, Girling A Change in mental health after smoking cessation: systematic review and meta-analysis. BMJ 2014;348:g1151. 24524926 10.1136/bmj.g1151 PMC3923980

[13] Lam E, Kvaavik E, Hamer M Association of secondhand smoke exposure with mental health in men and women: cross-sectional and prospective analyses using the U.K. Health and Lifestyle Survey. Eur Psychiatry 2013;28:276–81. 10.1016/j.eurpsy.2012.04.001 22959598

[14] Michal M, Wiltink J, Reiner I Association of mental distress with smoking status in the community: results from the Gutenberg Health Study. J Affect Disord 2013;146:355–60. 10.1016/j.jad.2012.09.019 23063238

[15] Bot M, Vink JM, Willemsen G Exposure to secondhand smoke and depression and anxiety: a report from two studies in the Netherlands. J Psychosom Res 2013;75:431–6. 10.1016/j.jpsychores.2013.08.016 24182631

[16] Nakata A, Takahashi M, Ikeda T Active and passive smoking and depression among Japanese workers. Prev Med 2008;46:451–6. 10.1016/j.ypmed.2008.01.024 18314186

[17] Wang S, Kou C, Liu Y Rural-urban differences in the prevalence of chronic disease in northeast China. Asia Pac J Public Health 2015;27:394–406. 10.1177/1010539514551200 25246500

[18] Schoenborn CA, Adams PE Health behaviors of adults: United States, 2005–2007. Vital Health Stat 10 2010:1–132. 245. 20669609

[19] Yang T, Yu L, Jiang S Household smoking restrictions among urban residents in China: individual and regional influences. Int J Public Health 2015;60:479–86. 10.1007/s00038-015-0672-0 25838120

[20] Cuéllar-Flores I, Sánchez-López MP, Limiñana-Gras RM The GHQ-12 for the assessment of psychological distress of family caregivers. Behav Med 2014;40:65–70. 10.1080/08964289.2013.847815 24754441

[21] Böhnke JR, Croudace TJ Calibrating well-being, quality of life and common mental disorder items: psychometric epidemiology in public mental health research. Br J Psychiatry 2015 10.1192/bjp.bp.115.165530 PMC4967770 26635327

[22] Huang C, Phillips MR, Zhang Y Malnutrition in early life and adult mental health: evidence from a natural experiment. Soc Sci Med 2013;97:259–66. 10.1016/j.socscimed.2012.09.051 23313495 PMC3726543

[23] Zhou X, Kang L, Sun X Prevalence and risk factors of post-traumatic stress disorder among adult survivors six months after the Wenchuan earthquake. Compr Psychiatry 2013;54:493–9. 10.1016/j.comppsych.2012.12.010 23337407

[24] Ballbè M, Martínez-Sánchez JM, Gual A Association of second-hand smoke exposure at home with psychological distress in the Spanish adult population. Addict Behav 2015;50:84–8. 10.1016/j.addbeh.2015.06.020 26111658

[25] Dachew BA, Fekadu A, Kisi T Psychological distress and associated factors among prisoners in North West Ethiopia: cross-sectional study. Int J Ment Health Syst 2015;9:39 10.1186/s13033-015-0033-7 26719761 PMC4696292

[26] Baillargeon J, Binswanger IA, Penn JV Psychiatric disorders and repeat incarcerations: the revolving prison door. Am J Psychiatry 2009;166:103–9. 10.1176/appi.ajp.2008.08030416 19047321

[27] Saxton JM, Scott EJ, Daley AJ Effects of an exercise and hypocaloric healthy eating intervention on indices of psychological health status, hypothalamic-pituitary-adrenal axis regulation and immune function after early-stage breast cancer: a randomised controlled trial. Breast Cancer Res 2014;16:R39 10.1186/bcr3643 24731917 PMC4052984

[28] Archer T, Josefsson T, Lindwall M Effects of physical exercise on depressive symptoms and biomarkers in depression. CNS Neurol Disord Drug Targets 2014;13:1640–53. 10.2174/1871527313666141130203245 25470398

[29] Bandiera FC, Arheart KL, Caban-Martinez AJ Secondhand smoke exposure and depressive symptoms. Psychosom Med 2010;72:68–72. 10.1097/PSY.0b013e3181c6c8b5 19949159 PMC2804907

[30] Sellini M, Sartori MP, Letizia C [Changes in the levels of ACTH and cortisol after passive exposure to cigarette smoke in smokers and non-smokers]. Boll Soc Ital Biol Sper 1989;65:365–9. 2550037

[31] Underner M, Perriot J, Peiffer G [Influence of tobacco smoking on the risk of developing asthma]. Rev Mal Respir 2015;32:110–37. 10.1016/j.rmr.2014.07.014 25765119

[32] Bao AM, Meynen G, Swaab DF The stress system in depression and neurodegeneration: focus on the human hypothalamus. Brain Res Rev 2008;57:531–53. 10.1016/j.brainresrev.2007.04.005 17524488

[33] Hamer M, Stamatakis E, Batty GD Objectively assessed secondhand smoke exposure and mental health in adults: cross-sectional and prospective evidence from the Scottish Health Survey. Arch Gen Psychiatry 2010;67:850–5. 10.1001/archgenpsychiatry.2010.76 20529994

[34] Kim NH, Choi H Secondhand smoke exposure and mental health problems in Korean adults. Epidemiol Health 2016;38:e2016009 10.4178/epih/e2016009 26988086 PMC4846743

[35] Mbulo L, Palipudi KM, Andes L Secondhand smoke exposure at home among one billion children in 21 countries: findings from the Global Adult Tobacco Survey (GATS). Tob Control 2016 Published Online First 11 Feb 2016. doi: 10.1136/tobaccocontrol-2015-052693 10.1136/tobaccocontrol-2015-052693 PMC5488799 26869598

[36] Tran TT, Yiengprugsawan V, Chinwong D Environmental tobacco smoke exposure and health disparities: 8-year longitudinal findings from a large cohort of Thai adults. BMC Public Health 2015;15:1217 10.1186/s12889-015-2547-y 26646160 PMC4673787

[37] Abdullah AS, Driezen P, Sansone G Correlates of exposure to secondhand smoke (SHS) at home among non-smoking adults in Bangladesh: findings from the ITC Bangladesh survey. BMC Pulm Med 2014;14:117 10.1186/1471-2466-14-117 25027238 PMC4107590

[38] Bell T, Watson M, Sharp D Factors associated with being a false positive on the General Health Questionnaire. Soc Psychiatry Psychiatr Epidemiol 2005;40:402–7. 10.1007/s00127-005-0881-6 15902411

