# Supplementary material for: Association between passive smoking and mental distress in adult never-smokers: a cross-sectional study
Source: BMJ Open. 2016 Jul 29;6(7):e011671. doi: 10.1136/bmjopen-2016-011671 (PMC4985981; doi:10.1136/bmjopen-2016-011671)
Supplement: Supplementary table [file bmjopen-2016-011671supp_table.pdf]

Supplementary Table 1.The result of Co linearity diagnostics

Co linearity diagnostics [a]

Model	Dimension	Eigenvalue	Condition Index	Variance proportions							
				Constant	Region	Gender	Age	Education	Occupation	Marriage status	Status of passive smoking
7	1	4.893	1.000	0.00	0.01	0.01	0.01	0.01	0.01	0.01	0.01
	2	0.925	2.300	0.00	0.00	0.06	0.03	0.01	0.00	0.73	0.00
	3	0.686	2.671	0.00	0.09	0.60	0.06	0.05	0.00	0.00	0.01
	4	0.661	2.721	0.00	0.03	0.27	0.20	0.05	0.02	0.18	0.01
	5	0.362	3.676	0.01	0.40	0.00	0.22	0.00	0.01	0.07	0.21
	6	0.217	4.749	0.00	0.32	0.02	0.31	0.53	0.21	0.00	0.00
	7	0.199	4.958	0.00	0.16	0.04	0.04	0.13	0.47	0.00	0.38
	8	0.058	9.202	0.99	0.00	0.01	0.14	0.22	0.27	0.02	0.38

a. Stepwise Regression was used in this analysis and the dependent variable: GHQ-12 scores
